# Radiological and clinical signatures to differentiate hepatocellular carcinoma from hepatoblastoma in children older than 5 years of age: a feasibility study

**DOI:** 10.1007/s00247-025-06190-w

**Published:** 2025-02-17

**Authors:** Gozde Ozer, H. Nursun Ozcan, Burak Ardicli, Tezer Kutluk, Berna Oguz, Mithat Haliloglu

**Affiliations:** Hacettepe University School of Medicine, Department of Radiology, Sıhhiye, 06100 Ankara, Turkey

**Keywords:** Liver neoplasms, Hepatoblastoma, Hepatocellular carcinoma, Pediatric, Computed tomography, Magnetic resonance imaging

## Abstract

**Background:**

Hepatoblastoma and hepatocellular carcinoma (HCC) are the most common primary malignant liver tumors in children. Although some characteristic imaging findings have been described in both hepatoblastoma and HCC, it is difficult to distinguish between these two tumors over the 5 years of age.

**Objective:**

To investigate clinical and radiological findings that may help differentiate hepatoblastoma and HCC over 5 years of age.

**Materials and methods:**

From 2007 to 2022, 19 consecutive patients older than 5 years old diagnosed with primary liver malignancy were yielded from our radiology archive retrospectively. Imaging features, age, sex, treatment, and follow-up data were recorded.

**Results:**

A total of 19 patients (16 boys; median age 7.5, min-max 5-17), ten HCCs and nine hepatoblastomas, were included. Serum alpha-fetoprotein (sAFP) values were significantly higher in hepatoblastoma patients (*n*=9), compared to the HCC (*n*=10) (*P*=0.002). Tumor size and PRETEXT stages were higher in hepatoblastoma patients; however, there was no statistical difference (*P*=0.06). Initial MRI was available for six patients with hepatoblastoma and seven patients with HCC, and there was no difference regarding ADCmin values.

**Conclusion:**

In the differential diagnosis of primary malignant liver tumor in a child older than 5 years of age, higher sAFP level may support the diagnosis of hepatoblastoma rather than HCC.

**Graphical Abstract:**

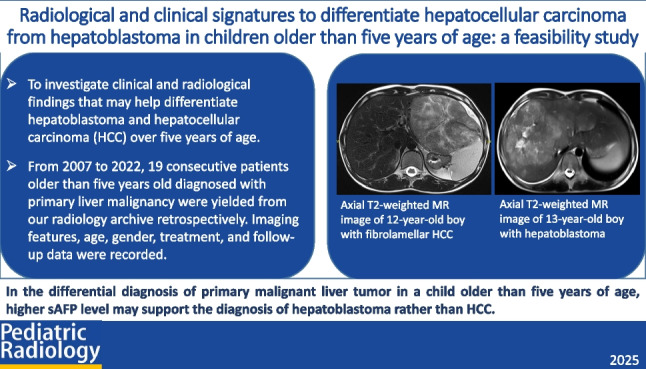

**Supplementary Information:**

The online version contains supplementary material available at 10.1007/s00247-025-06190-w.

## Introduction

Primary malignant tumors of the liver, which account for 1–2% of all pediatric malignancies, are relatively rare in children [1]. Two-thirds of pediatric liver tumors are malignant and almost half of these are hepatoblastomas. The median age at presentation of hepatoblastoma patients is 18 months, only 5% of patients are older than 4 years, and older age at diagnosis is associated with a poorer prognosis [2–5].

Hepatocellular carcinoma (HCC) is the second most common primary malignant liver tumor, often occurring after the age of 10 [6, 7]. The most common histologic subtypes of pediatric HCC are classic (73%) and fibrolamellar (25%) [8]. In rare cases, hepatocellular tumors may show histologic patterns of both HCC and hepatoblastoma. This entity is classified by the International Pediatric Liver Tumors Consensus Classification as a hepatocellular malignant neoplasm, not otherwise specified [9]. Therefore, pathologic diagnosis can be challenging due to the morphologic overlap between hepatoblastoma and HCC.

Imaging has three main goals when a liver tumor is suspected in a child: to confirm the presence of the tumor, to identify its extent, and, if possible, to characterize the tumor. When evaluating a liver mass, the most important information to guide the radiologist in the differential diagnosis are the patient’s age, the presence of predisposing factors and serum alpha-fetoprotein (sAFP) levels, and the imaging findings. In patients older than 5 years, it can be difficult to make this distinction. Due to the rarity of these tumors, it is understandable that there is still a paucity of data in the literature. The aim of this retrospective study is to evaluate the clinical and radiologic findings of hepatoblastoma and HCC patients older than 5 years, which may help in the differentiation.

## Materials and methods

### Patient selection

A retrospective consecutive study was performed in a tertiary pediatric hospital after the approval of our institutional review board (GO23/531). The archives in the pediatric radiology unit were reviewed for HCC and hepatoblastoma patients between 2007–2022. Thirteen HCC patients and 55 hepatoblastoma patients with pretreatment imaging were identified. Forty-eight patients under 5 years of age and one patient with pathologic diagnosis of hepatocellular malignant neoplasm-not otherwise specified were excluded from the study. A total of 19 patients (ten HCC, nine hepatoblastoma patients) at the initial diagnosis were included in our study. Initial computed tomography (CT) and magnetic resonance imaging (MRI) of all included patients were performed before any treatment, such as surgery, chemotherapy, or radiotherapy. In addition to imaging findings, histopathological results, age at diagnosis, sAFP levels, medical and surgical treatments, and follow-up data were recorded.

### Imaging technique

CT scans were performed using a GE LightSpeed 16-slice CT Scanner (GE Healthcare, Milwaukee, WI) after intravenous contrast administration on portal-venous phase in ten patients, and multiphasic post-contrast imaging in arterial, portal venous, and delayed venous phases were available for three patients. Axial images were reconstructed into 1.5–5-mm slice thickness with soft tissue algorithm. MRI scans were performed on using one of two scanners; 1.5-T Signa HDxt (GE Healthcare) and 3-T Signa Architect (GE Healthcare). Although there have been some changes in scanning parameters over the years, the routine imaging protocol in our institution included breath-hold coronal TRUE-FISP, axial T2-weighted half-Fourier acquisition single-shot turbo spin-echo, axial in- and opposed-phase chemical shift imaging, breath-hold T2-weighted fast spin-echo with fat saturation, and 3D T1-weighted gradient-recalled echo fat-suppressed sequences before and after injection of the contrast agent (Supplementary Material 1–2). Hepatobiliary phase images (at 20 min) were only available in two patients. Extracellular gadolinium-based contrast medium (Dotarem; Guerbet, Paris, France) at a dose of 0.2 mL/kg and hepatobiliary contrast medium (Primovist; Bayer Healthcare, Berlin, Germany) at a dose of 0.1 mL/kg were injected intravenously. In addition, patients were evaluated using DWI in axial planes with ADC mapping. A diffusion-weighted, multi-slice, single-shot spin echo-planar imaging sequence was performed. DWI was acquired with a diffusion-weighted factor using *b*-values of 0, 300, and 600 s/mm^2^ on the 1.5-T scanner and 50 and 800 s/mm^2^ on the 3-T scanner. A total of 14 preoperative MRIs and 13 preoperative CTs with different protocols were evaluated on our picture archiving and communication system (PACS; GE Healthcare).

### Radiologic evaluation

Pretreatment imaging features of 14 MRIs and 13 CTs were evaluated on the PACS by two pediatric radiologists with 11 years (H.N.O.) and 2 years of experience (G.O.) in pediatric body imaging. Six patients had only MRI, five patients had only CT, and eight patients had both MRI and CT preoperatively. Images were assessed for the following parameters: the presence of background liver disease, signal characteristics of lesions (T1-weighted; T2-weighted; arterial, venous, and delayed phases), presence of necrosis, central scar, tumor capsule, and tumor-in-vein. According to LIRADS criteria (Supplementary Material 3), arterial phase hyperenhancement (APHE), washout in the portal or delayed phase, and presence of tumor capsule were also noted. For the qualitative assessment of diffusion restriction, radiologists visually evaluated the signal intensity of the lesion compared to the background liver. Diffusion restriction was defined as hyperintensity on DWI with a high *b* value and hypointensity on the ADC map. For quantitative assessment, the largest possible regions of interest were manually traced by referencing T2-weighted and postcontrast T1-weighted images; ADCmin values were recorded. To assess tumor size, the maximum dimension of the tumor was measured in the transverse plane. Involved liver segments, pretreatment extent of disease (PRETEXT) stage, and annotation factors were also determined for each patient. Lymph node metastases were accepted as the short axis of the lymph node greater than 1.5 cm at the porta hepatis and greater than 1 cm elsewhere as defined by PRETEXT criteria [10].

### Statistical analysis

Descriptive analyses were presented using means and standard deviations for normally distributed and medians and min-max for the non-normally distributed. Fisher’s exact test was used to determine whether there was a significant relationship between categorical variables. Mann-Whitney *U* test was used for nonparametric data. Analyses were performed with the SPSS 28.0 (IBM, Armonk, NY) software program. The statistical significance level was determined as *P*<0.05.

## Results

A total of 19 patients (ten HCC, nine hepatoblastoma), three girls and 16 boys, with a median age 7.5 (min-max 5–17), were included in our study.

### Hepatocellular carcinoma

Ten patients with HCC (8 boys; median age 12.5, min-max 5–15) were included. Demographic and clinical findings of HCC patients are shown in Table 1. Background liver parenchyma was normal in eight patients (80%). Radiological findings of chronic liver parenchymal disease were detected in two patients, one had cryptogenic cirrhosis and one had hepatitis B virus infection. Initial sAFP levels were increased in six patients. The median sAFP level at diagnosis was 104 ng/ml (min-max 1.18–328,259 ng/ml). Fibrolamellar HCC was detected in five patients, and two had elevated sAFP levels. In addition, one patient with classical type HCC had normal sAFP levels and tyrosinemia type 1. Follow-up data were available in nine patients with a median follow-up time of 27 months (min-max 2–84 months); one patient applied to another institution for treatment after diagnosis. One patient had a diffuse tumor involving the entire parenchyma at the time of diagnosis, and the patient died 2 months after the diagnosis. Another deceased patient was considered inoperative at the diagnosis, and his lesions increased under chemotherapy treatment. The remaining seven patients were followed up as alive and well. A total of six patients underwent surgical treatment, and liver transplantation was performed in one patient who had fibrolamellar HCC due to the development of multiple recurrent tumors after surgical resection. Liver transplantation was performed on one patient diagnosed with tyrosinemia type 1.

**Table 1:** The demographic and clinical findings of the hepatocellular carcinoma patients

Patient no	Age at diagnosis	Gender	Pathologic diagnosis	sAFP (ng/ml) (0–100 ng/ml)	Primary diagnoses/background liver	FU periods (months)	Treatment/recurrence/transplantation
1	12	M	Fibrolamellar HCC	6,578	-/N	27	Surgery and chemotherapy / + / +
2	5	M	Fibrolamellar HCC	1.18	-/N	50	Surgery / - / -
3	15	F	Fibrolamellar HCC	3.77	-/N	84	Surgery and chemotherapy /- / -
4	14	M	HCC	5.89	Tyrosinemia type 1/N	22	- / - / +
5	8	M	HCC	75.3	-/cirrhosis	2	Chemotherapy /- / -
6	14	M	Fibrolamellar HCC	2.34	-/N	2	Surgery / - / -
7	15	M	Fibrolamellar HCC	134	-/N	72	Surgery /- / -
8	5	M	HCC	2,382	Tyrosinemia type 1/N	NA	NA
9	9	F	HCC	328,259	HBV(+)/N	13	Surgery and chemotherapy /- / -
10	13	M	HCC	2,658	HBV(+)/cirrhosis	25	Chemotherapy /+/ -

*F* female, *M* male, *HCC* hepatocellular carcinoma, *N* normal (background liver parenchyma), *NA* not applicable

**Fig. 1:**
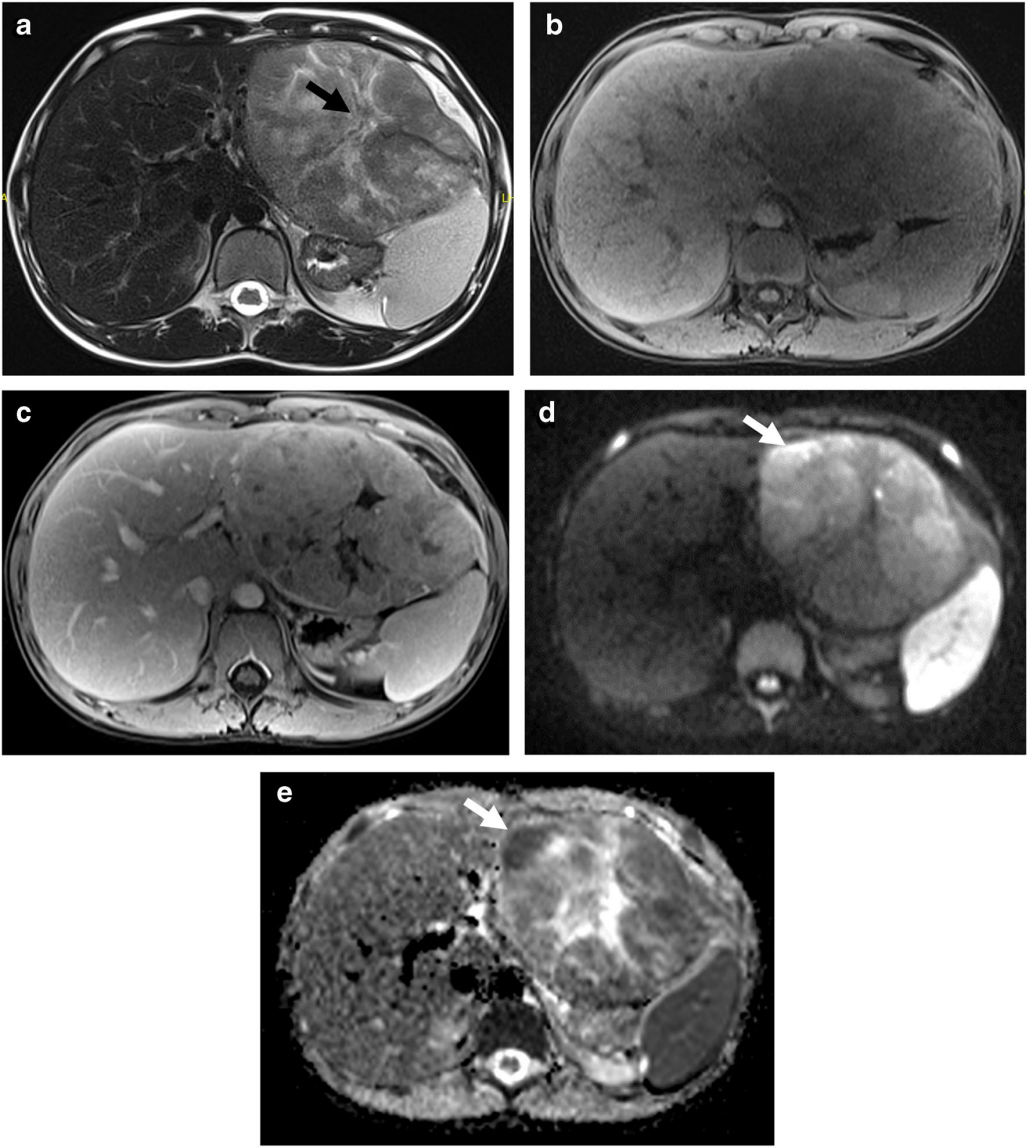
Fibrolamellar hepatocellular carcinoma in a 12-year-old boy. **a**–**c** Axial T2-weighted (**a**), and precontrast (**b**) and postcontrast (**c**) T1-weighted images show large heterogeneous mass (*arrow* in **a**) in the left liver lobe with central scar. **d**, **e** Axial diffusion weighted image at *b*=800 s/mm^2^ (**a**) and ADC map (**b**) demonstrate restricted diffusion (*arrows*)

CT and MRI findings of each patient are shown in Table 2. The tumor size ranged from 21 mm to 153 mm, with a median tumor size of 97 mm. Tumor necrosis was detected in seven patients (70%), tumor in vein detected in three patients (30%), central scar in four patients (with fibrolamellar HCC) (40%) (Fig. 1), and tumor capsule in two patients (20%). Intralesional fat was detected in one patient on dual-phase imaging. Postcontrast multiphasic imaging was not performed in one patient, and APHE and washout were detected in 5/9 patients (66%). In two patients where hepatobiliary contrast material was used, tumors were detected as hypointense in the hepatobiliary phase. DWI was available for seven patients, and diffusion restriction was detected in all but one lesion by qualitative assessment. ADCmin values ranged from 0.7×10^−3^ mm^2^/s to 1.7×10^−3^ mm^2^/s (ADCmin median 1×10^−3^ mm^2^/s) by quantitative assessment. PRETEXT stages were distributed as PRETEXT I in three patients (30%), PRETEXT II in four patients (40%), and PRETEXT IV in three patients (30%).

**Table 2:** Imaging findings and PRETEXT staging of the hepatocellular carcinoma patients

Patient no.	Tumor size (mm)	Necrosis/central scar/capsule	Washout	ADCmin (×10^−3^ mm^2^/s)	PRETEXT	Annotation factors
1	120	+ / + / +	+	0.7	I	-
2	21	- / - / -	+	1.7	I	-
3	130	+ / + / -	-	1	II	V, N
4	24	- / - / -	+	0.8	II	N
5	95	+ / - / -	NA	1.1	IV	V, P, F, C, N, E
6	100	+/ + / -	-	NA	II	V, N
7	85	+ / + / -	-	1.2	II	N
8	30	- / - / +	+	0.9	I	-
9	153	+/ - / -	-	NA	IV	V, P, C, N, E, M
10	112	+ / - / -	+	NA	IV	V, P, N

*C* caudate involvement, *E* extrahepatic spread, *F* multifocal tumor, *M* distant metastases, *N* lymph node involvement, *NA* not available, *P* portal vein involvement, *R* tumor rupture, *V* hepatic venous/inferior vena cava involvement

### Hepatoblastoma

Nine hepatoblastoma patients’ (8 boys; median age 7, min-max 5–12) demographic and clinical findings are shown in Table 3. All patients had normal liver parenchyma. sAFP levels were increased in all patients at diagnosis. The median sAFP level at diagnosis was 66,688 ng/ml (min-max 21,105–765,000 ng/ml). Follow-up data were available in all patients with a median follow-up time of 26 months (min-max 12–80). Two patients have died 23 and 61 months after the hepatoblastoma diagnosis. The remaining seven patients were followed up as alive and well. A total of four patients underwent surgical treatment, and three patients underwent liver transplantation without primary resection of the tumor.

**Table 3:** The demographic and clinical findings of the hepatoblastoma patients

Patient no.	Age at diagnosis	Gender	Pathologic diagnosis	sAFP (ng/ml) (0–100 ng/ml)	Primary diagnoses/background liver	FU periods (months)	Treatment/recurrence/transplantation
1	7	M	Epithelial HB, embryonal	239,855	-/N	24	- / - / +
2	16	M	Epithelial HB, embryonal	21,105	-/N	26	Chemotherapy/-/-
3	17	M	Epithelial HB, embryonal	66,688	X-linked lymphoproliferative disease/N	23	Surgery and chemotherapy/+/-
4	7	M	Mixed HB	37,241	-/N	14	Surgery and chemotherapy/+/-
5	5	M	Epithelial HB, embryonal	224,887	-/N	72	Chemotherapy/-/+
6	13	M	Epithelial HB, fetal and embryonal	706,729	-/N	35	- / + / +
7	6	M	Epithelial HB, embryonal	263,000	-/N	12	Chemotherapy /-/ -
8	5	F	Epithelial HB, fetal	765,000	-/N	80	Surgery and chemotherapy/-/ -
9	6	M	Epithelial HB, fetal and embryonal	27,811	-/N	61	Surgery and chemotherapy /-/ -

*F* female, *HB* hepatoblastoma, *M* male, *N* normal (background liver parenchyma), *NA* not applicable

**Fig. 2:**
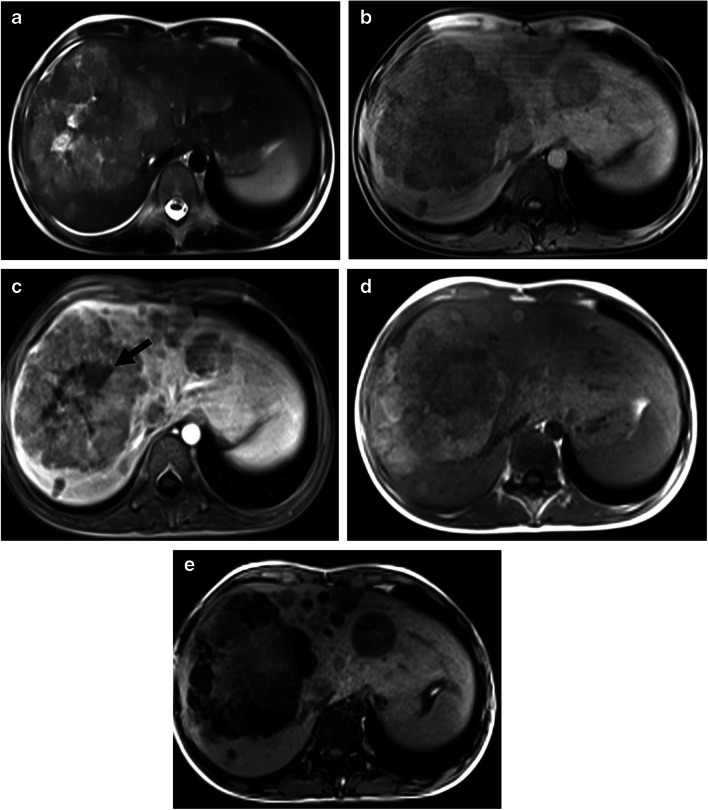
Hepatoblastoma in a 13-year-old boy. **a**–**c** Axial T2-weighted (**a**), and precontrast (**b**) and postcontrast (**c**) T1-weighted images show multifocal tumor with necrosis (*arrow*) and heterogenous enhancement. **d**, **e** Dual-echo in-phase (**d**) and out-of-phase (**e**) GRE T1-weighted images show signal loss on out-of-phase due to intralesional fat

CT and MRI findings of each patient are shown in Table 4. The tumor size ranged from 97 mm to 215 mm, with a median tumor size of 120 mm. Tumor necrosis was detected in all nine patients (100%), and central scar in two patients (40%), and tumor in vein in one patient (11%). Intralesional fat was detected in three patients on dual-phase imaging (Fig. 2). DWI was available for six patients, and diffusion restriction was detected in all lesions by qualitative assessment. ADC values ranged from 0.5×10^−3^ mm^2^/s to 1.5×10^−3^ mm^2^/s (ADCmin median 0.7×10^−3^ mm^2^/s). PRETEXT stages were distributed as PRETEXT II in one patient (11%), PRETEXT III in two patients (22%), and PRETEXT IV in six (66%) patients.

**Table 4:** Imaging findings and PRETEXT staging of the hepatoblastoma patients

Patient no.	Tumor size (mm)	Necrosis/central scar/capsule	ADCmin (×10^−3^ mm^2^/s)	PRETEXT	Annotation factors
1	102	+ / - / -	0.5	IV	E, F
2	215	+/ - / -	1.5	IV	V, P, F, C, N
3	105	+ / - / -	0.9	III	V, F
4	97	+ / - / -	0.5	II	N
5	100	+ /- / -	0.6	IV	V, P, F,
6	120	+ / - / -	0.9	IV	V, P, F, C
7	155	+ / - / -	NA	IV	V, P, F, C, N, M
8	176	+ / - / -	NA	III	V, P
9	130	+ / - / -	NA	IV	V, P, N, M

*C* caudate involvement, *E* extrahepatic spread, *F* multifocal tumor, *M* distant metastases, *N* lymph node involvement, *NA* not available, *P* portal vein involvement, *R* tumor rupture, *V* hepatic venous/inferior vena cava involvement

### Comparison of hepatocellular carcinoma and hepatoblastoma groups

The comparison of the two groups in terms of clinical and radiological findings is given in Table 5. There was no significant difference between hepatoblastoma and HCC groups in terms of age (*P*=0.46) and sex (*P*=1). sAFP values were significantly higher in the hepatoblastoma group compared to the HCC group (*P*=0.002). Tumor size was higher in the hepatoblastoma group than in the HCC group; however, albeit, this failed to reach statistical significance (*P*=0.06). Although PRETEXT stages III and IV were more common in the hepatoblastoma group (*n*=2 and *n*=6, respectively), and stages I and II were more common in the HCC group (*n*=3 and *n*=4, respectively), there was no statistical difference between groups. APHE (55%) and washout (55%) were only detected in the HCC group. Also, tumor necrosis was found in most patients, and there was no difference between the two groups. Initial MRI was available for six patients with hepatoblastoma and seven patients with HCC, and there was no difference regarding ADCmin values (*P*=0.13).

**Table 5:** Comparison of clinical and imaging findings of hepatoblastoma and hepatocellular carcinoma patients over 5 years of age

	HCC (*n*=10)	Hepatoblastoma (*n*=9)	*P-*value
Age at diagnosis, median	12.5	7	0.46
Gender (F/M)	2/8	1/8	1
sAFP levels (ng/ml), median	104	66,688	0.002*
Maximum tumor size (mm), median	97	120	0.06
Tumor in vein	3 (30)	1 (11)	0.582
Multifocal tumor	3 (30)	5 (55)	0.37
Tumor necrosis	7 (70)	9 (100)	0.21
Central scar	4 (40)	3 (33)	1
Intralesional fat	1 (10)	3 (33)	0.303
ADCmin (mm^2^/s), median	1×10^−3^	0.7×10^−3^	0.13

Data in parentheses represent the number of patients in percentage

*F* female, *M* male, *HCC* hepatocellular carcinoma, *sAFP* serum alpha fetoprotein

*P < 0.05

## Discussion

The main finding of our study is that hepatoblastoma patients (>5 years old) have higher sAFP levels compared to HCC patients. High sAFP levels are a typical laboratory finding in hepatoblastoma and HCC patients [8, 11]. AFP is a glycoprotein that is normally produced by the yolk sac and fetal liver during pregnancy, and serum levels decline rapidly after birth due to suppression of AFP expression [12]. In our study, higher sAFP levels in hepatoblastoma may be explained by the fetal and/or embryonic origin of the tumor. Unlike hepatoblastoma, in HCC, the tumor tissue regains the ability to produce oncofetal protein as a result of complex genetic mechanisms that have not yet been fully elucidated [12]. To the best of our knowledge, there is no study comparing HCC and hepatoblastoma in terms of sAFP levels.

It is known that the sAFP levels are usually normal in fibrolamellar HCC and have a good prognosis compared to classical type HCC in adults [13]. However, Katzenstein et al. and Wahab et al. showed that fibrolamellar HCC may not be associated with a favorable prognosis in children, and elevated sAFP levels of up to 10% have been reported [14, 15]. Also, another study by McDonald et al. showed that 44% of adult fibrolamellar HCC patients have elevated sAFP levels and higher levels are associated with worse prognosis [13]. In our study, a fibrolamellar HCC patient with a high sAFP level developed multiple recurrent tumors after surgical resection, and liver transplantation was performed. In contrast to HCC, low sAFP levels at diagnosis (<100 ng/ml) are considered high risk in patients with hepatoblastoma and are mostly associated with small cell undifferentiated histologic type [11]. None of the patients in our cohort had low sAFP levels, which may be explained by epithelial or mixed histological type of all tumors.

In the literature, both hepatoblastoma and HCC patients had slight male predilection [16, 17]. Similarly, in our study, most of the patients were male in both groups.

The PRETEXT staging system is routinely used for staging, risk stratification, and determining surgical approach in children with primary hepatic malignancies [10, 18]. In our study, PRETEXT III (22%) and IV (66%) tumors were more common in the hepatoblastoma group. Hiyama et al. and Meyer et al. reported that PRETEXT stage IV has a rate of 15–23% in hepatoblastoma [19, 20]. In comparison to these results, we found that PRETEXT IV disease was more common in patients older than 5; however, it may not be possible to extrapolate this finding due to the small sample size. Consistent with the literature by D’Souza et al., PRETEXT I and II tumors were more frequent in the HCC group [21]. A recently published article by Rees et al. showed that HCC patients with predispositions tend to have smaller tumor size, lower PRETEXT stage, and less frequent tumor in vein [22]. This could be explained by surveillance imaging in children with predisposing factors. The two HCC patients with tyrosinemia in our study were detected at routine follow-up imaging and classified as PRETEXT I and II. Annotation factors including hepatic venous/vena cava inferior involvement (V), portal venous involvement (P), extrahepatic disease contiguous with the primary liver tumor (E), multifocal tumor (F), tumor rupture (R), involvement of the caudate lobe (C), lymph node metastasis (N), and distant metastasis (M) were associated with adverse prognosis [23]. The most common positive annotation factor was hepatic venous/vena cava inferior involvement in both groups (50% in the HCC group and 77% in the hepatoblastoma group). In addition, portal venous involvement and multifocal tumor were rare in HCC patients in our cohort when compared with the literature [24]).

Liver Imaging Reporting and Data System (LI-RADS) provides a standardized lexicon for radiologic imaging and is commonly used to diagnose adult HCC. However, LI-RADS major criteria have a limited value in children due to low specificity and interobserver agreement (24). Similar to the results by Khanna et al., we found APHE 55%, non-peripheral washout 55%, and enhancing capsule 22% in the HCC group [25]. APHE and washout were not detected in any of the hepatoblastoma cases. Therefore, the LI-RADS major criteria, although nonspecific, may help distinguish these two tumors. Finally, we found a signal drop on dual-phase GRE T1-weighted images representing intralesional fat in three patients with hepatoblastoma. The presence of fat in a malignant liver lesion is considered a quite specific but insensitive imaging feature of HCC in adults [26]. However, intralesional fat is not a typical finding of either pediatric HCC or hepatoblastoma.

Our study has several limitations. First, due to the retrospective design and long time span of the study, not all imaging modalities were available for each patient, and the image acquisition technique varied widely. Second, although HCC and hepatoblastoma are rare tumors in children over 5 years old, the overall sample size was small. The lack of a similar previous study also prevented us from conducting a power analysis. Although tumor size and PRETEXT stages were higher in hepatoblastoma patients, these differences were not statistically significant, likely due to the limited sample size. Further definitive studies with larger patient groups are needed to identify clinical and radiological signatures that may be helpful in the differential diagnosis of primary malignant liver tumors in children over the age of 5 years old.

In conclusion, although hepatoblastoma is rare in patients over 5 years of age, it should be included in the differential diagnosis of radiologically malignant tumors beyond HCC. This study indicates that high AFP levels may serve as a laboratory finding to support the diagnosis of hepatoblastoma in this age group. These observations require confirmation from other cohorts.

## Supplementary Information

Below is the link to the electronic supplementary material.

Supplementary file1 (DOCX 20 KB)

## Data Availability

No datasets were generated or analysed during the current study.
