# Supplementary material for: Radiological and clinical signatures to differentiate hepatocellular carcinoma from hepatoblastoma in children older than 5 years of age: a feasibility study
Source: Pediatr Radiol. 2025 Feb 17;55(5):946–54. doi: 10.1007/s00247-025-06190-w (PMC12065736; doi:10.1007/s00247-025-06190-w)
Supplement: Supplementary file 1 — Supplementary file1 (DOCX 20 KB) [file 247_2025_6190_MOESM1_ESM.docx]

**Table S1.** MRI sequence parameters for 1.5T MRI

| Sequence | Plane | TR (ms) | TE (ms) | Flip angle | Fat saturation | FOV (mm) | Slice thickness (mm) |
| --- | --- | --- | --- | --- | --- | --- | --- |
| T2 SSFP | Coronal | 4.3 | 2.1 | 60 | - | 350×350 | 4.5 |
| T1 GRE in and out of phase | Axial | 160 | In phase:4.9 Out of phase:2.4 | 70 | - | 350×262 | 6 |
| T2 FSE FS | Axial | 3050 | 125 | 150 | + | 350×350 | 6 |
| T1 3D GRE LAVA | Axial | 5 | 2.4 | 10 | + | 350×266 | 3 |
| T1 3D GRE LAVA hepatobiliary | Axial | 5 | 2.4 | 30 | + | 350×266 | 3 |
| Diffusion (b=0, 300, 600 s/mm^2^) | Axial | 6438 | 87 | 90 | - | 350×288 | 6 |

**Table S2**. MRI sequence parameters for 3T MRI

| Sequence | Plane | TR (ms) | TE (ms) | Flip angle | Fat saturation | FOV (mm) | Slice thickness (mm) |
| --- | --- | --- | --- | --- | --- | --- | --- |
| T2 FSE BH | Coronal | 800 | Min | 90 | - | 420 | 4 |
| T1 GRE in and out of phase | Axial | 150 | In phase:4.9 Out of phase:2.4 | 45 | - | 350x300 | 6 |
| T2 FSE FS | Axial | 800 | Min | 90 | + | 350x300 | 5 |
| T1 3D GRE LAVA | Axial | 4.3 | 1.8 | 12 | + | 350x263 | 2.2 |
| T1 3D GRE LAVA hepatobiliary | Axial | 4.3 | 1.8 | 25 | + | 350x263 | 2.2 |
| Diffusion (b=50, 800 s/mm^2^) | Axial | 5300 | 59 | 90 | - | 340x163 | 6 |

**Table S3**. LIRADS criteria

| **Major criteria** | **Definiton** |
| --- | --- |
| Arterial phase hyperenhancement (APHE) | Non-rim arterial hyperenhancement of a lesion which is greater than the enhancement of the adjacent liver |
| Non-peripheral washout | Decrease in attenuation or intensity from earlier to later phase, resulting in hypoenhancement in the portal venous or delayed phase |
| Capsule | Peripheral rim of smooth hyperenhancement seen in the portal venous phase, transitional phase, or delayed phase |
| Size | A large lesion has a greater chance of being a HCC than a small lesion |
| Threshold growth | diameter increase ≥50% increase in ≤6 months on follow-up imaging |
